# SA-Mediated Regulation and Control of Abiotic Stress Tolerance in Rice

**DOI:** 10.3390/ijms22115591

**Published:** 2021-05-25

**Authors:** Kalaivani Nadarajah, Nur Wahida Abdul Hamid, Nur Sabrina Natasha Abdul Rahman

**Affiliations:** Department of Biological Sciences and Biotechnology, Faculty of Science and Technology, Universiti Kebangsaan Malaysia, Bangi 43,600, Selangor, Malaysia; nurwahida9827@gmail.com (N.W.A.H.); nashabreena@yahoo.com (N.S.N.A.R.)

**Keywords:** salicylic acid, abiotic stress, modulation, repression, phytohormone

## Abstract

Environmental or abiotic stresses are a common threat that remains a constant and common challenge to all plants. These threats whether singular or in combination can have devastating effects on plants. As a semiaquatic plant, rice succumbs to the same threats. Here we systematically look into the involvement of salicylic acid (SA) in the regulation of abiotic stress in rice. Studies have shown that the level of endogenous salicylic acid (SA) is high in rice compared to any other plant species. The reason behind this elevated level and the contribution of this molecule towards abiotic stress management and other underlying mechanisms remains poorly understood in rice. In this review we will address various abiotic stresses that affect the biochemistry and physiology of rice and the role played by SA in its regulation. Further, this review will elucidate the potential mechanisms that control SA-mediated stress tolerance in rice, leading to future prospects and direction for investigation.

## 1. Introducing SA as a Mitigator of Abiotic Stress in Rice

Agricultural systems are open to both abiotic and biotic stresses. Abiotic stresses encompass so many diverse stresses and their significance and relevance to agricultural productivity is paramount. One of the main reasons for the degradation of our agricultural systems is due to the application of anthropogenic activities, which has resulted in abiotic stresses such as high metal content in soil, nutrient depletion, salinity, and changes to the physico-chemical structure of the soil [1,2]. The impact of abiotic stresses on plant biochemistry and physiology have direct consequences on growth, development and yield, where it transcends all developmental stages from seed germination to maturity. The overall effect of abiotic stress to the yield component of rice can be devastating and losses can sometimes reach 70% of expected yield [3].

As an agriculturally important crop, rice remains one of the most important staple foods worldwide and is grown in many countries to meet both their local and export demands. Biotic and abiotic stresses affect rice yield. Further, there is a need for more agricultural land, where the pressure for increased production has pushed rice farming into marginalized land. These pieces of land suffer from poor irrigation, salinity, metal toxicity, and nutrient deficiency. Therefore, the quest towards sustainable development will include identifying ways and means by which resistant or tolerant rice varieties may be raised against these abiotic stresses. One way of identifying these resistance traits is by studying the defense mechanism of plants. Several studies have implicated the involvement of growth regulators in resistance and defense signaling of plants including rice [4,5]. One molecule that has a significant role to play in resistance and defense signaling is salicylic acid (SA).

SA, an endogenous signal molecule, regulates plant responses and serves as a signal transducer. It provides protection against both abiotic and biotic stresses and controls processes such as antioxidant defense, nitrogen metabolism, photosynthesis, water stress and others in order to protect the plant cells from accumulation of toxic compounds and cell death [6]. However, the ability of this molecule to regulate the entire process of defense and resistance is still poorly understood, especially in rice. Factors that contribute towards this complexity are the variation in the level of SA in different plant species and the impact of environment on endogenous SA levels. The impact of endogenous SA levels on rice and other plant species are dependent on developmental stages and the concentration of SA used in the experiment. In addition to concentration, SA is regulated in a spatio-temporal manner in plants [7,8]. The highest level of endogenous SA has been reported in rice compared to other plants, where the content in tissue is several folds higher than that reported in other model plant systems, and cereals. Typically the content of SA in rice is between 5 and 30 µg/g fresh weights, compared to 1 µg/g in other plant systems [9,10]. Further the application of exogenous SA through imbibition and spraying induces abiotic stress tolerance towards drought, cold, heavy metal, osmotic and salt stress tolerance in rice and other plant species [11,12,13,14,15,16]. At the molecular level, abiotic stress tolerance is known to induce several genes in plants, and most of these genes are linked to SA-dependent activation. These genes include chaperones, antioxidants, secondary metabolites and other stress related proteins [17,18,19].

Although there are many studies that have been conducted on the effect of SA in abiotic stresses in plants, the information of its role in abiotic stress tolerance in rice has been limited. Therefore, in this review we endeavor to present accounts on SA biosynthesis and metabolism, the role played by SA in different abiotic stresses as well as the potential underlying mechanisms ordering SA-mediated abiotic stress tolerance in rice.

### Biosynthesis and Metabolism Salicylic Acid

Salicylic acid (SA) is synthesized in plants through two different pathways: the phenylpropanoid and the isochorismate pathways [20]. Biosynthesis of SA was initially studied biochemically in tobacco leaves, leading to the discovery of the cytoplasmic phenylpropanoid pathway [21]. The conversion of phenylalanine to trans-cinnamic acid (t-CA), is catalyzed by phenylalanine ammonia-lyase (PAL). t-CA is then converted to benzoic acid (BA), and SA is derived from BA hydroxylation and catalyzed by benzoic acid 2-hydroxylase (BA2H) [22]. Wildermuth et al. [23] reported a SA pathway that ran from chorismate through to isochorismate in pathogen-infected *Arabidopsis*, which leads to the identification of two putative *isochorismate synthase* (*ICS*) genes. More studies in Arabidopsis revealed that SA could also be synthesized from chorismate to isochorismate. Isochorismate pyruvate lyase (IPL) converted isochorismate to SA. Catinot et al. [24] also reported that the production of SA in response to biotic and abiotic stress depended on the isochorismate in *Nicotiana benthamiana* [22,25]. In rice, however, oHCA has been implicated more than isochorismate. This will be discussed in detail in the following sections.

## 2. The Influence of Salicylic acid on Abiotic Stress Tolerance in Rice

### 2.1. SA’s Influence on Salinity Stress

As a complicated stress, salinity results in osmotic stress, nutrient deficiency, physiological and biochemical damage that hampers growth and development [26] especially of crops that grow near coastal areas and estuaries. Rice is grown in reclaimed land, most often near the coasts and estuaries. Studies conducted show that the application of SA is able to condition the plant to produce better yield under salt stress. When SA is administered to rice during germination in salt stress, it significantly increased shoot and root lengths causing increased tolerance salinity. These findings are similar to that reported by Asadi et al. [27], Boukraâ et al. [28], Lee et al. [29], and Torabian [30], where SA pretreatment induced germination levels under salt stress. Treatment with SA in the vegetative stage of rice rescued the plants from hampered growth and development. Similar findings were also reported in other studies [31] where in *Vicia faba* L., SA improved photosynthesis and antioxidant response in planta [32]. SA also enhanced growth in other cereals under salt stress such as barley, wheat and maize [33,34,35]. SA has also been shown to increase yield components in ASD16 and BR26 rice lines. This contribution to yield may be as a consequence of SA’s involvement in ion movement, flowering and photosynthesis [31,36].

SA is able to reduce the NA^+^ and Cl^−^ levels in the cell in salt stress. The exogenous SA application induces internal SA levels that can cause physiological changes in the plant. In Arabidopsis, exogenous SA reversed the effect of salt and oxidative stresses observed in seedling germination and development [37]. SA application increased K^+^ content which reduced Na^+^ levels in *Arabidopsis* and mung bean under salt stress [38,39]. During salt stress, rice grains exhibited lower content of carbohydrate and proteins which were important components for growth and development [40]. A high affinity K+ Transporter, OsHKT1, is involved in assisting with coping against salt stress where this molecule is found upregulated in the phloem during stress. OsMYBc binds to OsHKT1 promoter to induce expression. Knockout mutants of OsMYBc showed reduced salt tolerance implying a role for this complex in salt stress tolerance of rice.

By applying SA, SA responsive genes are activated to moderate physiological processes to restore normal growth and development [41]. While application of SA during vegetative and germination restores the plant to reduced stress, SA in reproductive stage did not improve the morphological characters and yield. However, in response to SA treatment *OsCM*, *OsICS* and *OsPAL* genes are induced in response to salt stress in rice. In addition to these genes, enzymes such as superoxide dismutase (SOD), peroxidase (POX), glutathione reductase (GR) and catalase (CAT) were elevated during salt stress. However, when SA was applied, these levels reduced within the cells and therefore restored physiological mechanisms in rice plants [40,42]. This implies that the application of SA induced antioxidant defense response to protect the plant against stress [43]. The levels of these enzymes, however, are dependent on the developmental stage, stress level, duration and metabolic status of plants [40].

SA reduced NaCl stress on *Hordeum vulgare* through decreased cellular malondialdehyde (MDA) and ROS production [44]. Through SA priming it may be possible to enhance production of major glutathione (GSH) such as glutathione S-transferase GST to metabolize H_2_O_2_. In tomatoes, salinity stress has been mitigated through SA. This has resulted in changes of expression patterns in the GST family [45]. Furthermore in wheat and rice (*Oryza sativa* spp. Japonica), exogenously SA treated plants showed improved expression in transcripts of various antioxidant components (dehydroascorbate reductase (*DHAR*), glutathione peroxidase (*GPX1*, *GPX2*), glutathione reductase (*GR*), glutathione synthetase (*GS*), glutathione S-transferase (*GST1*, *GST2*), monodehydroascorbate reductase (*MDHAR*)) under salt stress [46]. In addition, SA is able to restore the membrane potential and control salt induced losses of potassium through the GORK channel, and result in salt tolerance in *A. thaliana* [38].

### 2.2. SA’s Influence on Drought Stress

Drought influences rice by adversely affecting the plant weight, the process of photosynthesis, stomatal conductance, water relations in the plant, and starch metabolism [47,48]. Different studies showed a positive effect in SA application on photosynthesis in drought stress induced plants [49]. SA application in drought stressed wheat resulted in enhanced photosynthesis and rubisco activity [49]. In rice the application of SA significantly reduced membrane electrolyte leakage, hydrogen peroxide accumulation and MDA content build-up [50]. Drought stress results in the accumulation of ROS causing damage to cell and DNA, therefore affecting the normal function of the organism [6,51].

In a study conducted by Dhawan et al. (2021) [52] rice genotypes with varying drought tolerance were used to determine the relationship between the SA metabolism and protection afforded. When SA was applied to foliar tissue and used in seed treatment of Basmati 2000, improved performance under drought stress was observed. There was no increase in expression levels of chorismate synthase and isochorismate synthase genes in response to SA treatment. These genes are linked to SA biosynthesis and metabolism. Studies have shown that there is a correlation between ortho-hydroxy-cinnamic (oHCA) and SA indicating that in rice SA production is likely through oHCA and not chorismate pathway. It is believed that the chorismate pathway produced basic SA levels compared to the higher levels seen in rice [52,53].

When SA was applied on rice foliar tissue, drought resistance in rice was elevated. Many studies showed no significant change in rice SA level even with stresses such as drought. Polyamines (PA) were reported to play a role in responding to stresses in plants. While SA levels did not change much in stress, PA levels were however elevated in environmental stress [53]. This, however, showed that SA and PA are not correlated. SA was also genotype and environmentally modulated in rice [54]. While there was no correlation between SA levels in rice during stress and production of GTR enzyme, there was elevated levels of GST produced in the tissues of drought stressed rice. SA therefore may play a role in acclimatization in rice through enzymatic mechanisms [55,56].

In drought stressed rice seedlings, SA application induced antioxidant enzymatic activity. This includes the increase in enzymes like SOD, peroxidases, and CATs. Though CATs have been inhibited in other plants like tobacco, in rice (*Oryza sativa* var Teqing) these were elevated in certain isoenzymes [57]. CATa was elevated and CATb is inhibited in rice [58]. Through the production of antioxidants, ROS damage was reduced, and this is seen from the effect on membrane permeability. The activation of antioxidants by SA improved the overall membrane integrity and therefore reduced loss of water from rice tissues; maintaining proper photosynthesis and general metabolism. Drought significantly reduced photosynthesis and stomatal conductance, thus affecting growth and yield of crop [59]. In *H. vulgare*, supplementation with SA (500 μM) in drought resulted in increased net CO_2_ assimilation as a consequence of increased stomatal conductance that resulted in increased plant biomass [60]. In addition to induction of antioxidant enzymes in response to SA, exogenous SA also resulted in the induction of other enzymes like MDHAR, DHAR GR GSH, GPX and GSH. These enzymatic and non-enzymatic components play a role in moderating drought stress [61]. Foliar application showed better induction of antioxidants compared to seed treatment. This was especially observed in induced antioxidant defense in drought tolerant *Zea mays* [62]. In SA treated *T. aestivum*, less wilting and better plant height and weight were observed [63].

Studies conducted on cereals showed that there was a correlation between gene expression and SA treatment under drought stress condition. When treated with SA, drought exposed *T. aestivum* exhibited enhanced expression of GST, GR and MDHAR transcripts [64]. Further, SA mutants (*acd6* and *cpr5*) exhibited control over stomatal closure and drought tolerance in *A. thaliana*, which is mediated through SA induced PR expression [65]. In *T aestivum*, many proteins that are involved in physiological functions were identified including those that were involved in abiotic stress management [63]. Most studies showed that SA when applied at different concentrations showed varying effects on the plants and their physiological and metabolic activities. SIZ1 is a positive regulator of drought stress tolerance. SIZ1 mutants of Arabidopsis responded to elevated levels of SA through higher expressions of *PR1* (Pathogenesis Related gene 1) and increased sensitivity to phosphorus-limited conditions. Similarly, rice homologs in response to SA and cytokinins directly regulated plant growth and development [66]. In *A. thaliana*, SA-mediated SIZ1 activity regulated stomatal closure and enhanced drought tolerance [44]. Similar effects were seen in rice.

### 2.3. SA’s Effect on Temperature Stress

Temperature fluctuations as a result of climate change have been implicated as a potential environmental stress to plants. Environmental temperature physiologically and biochemically affect plants and directly affects gene expression and molecular mechanisms [59,67]. High temperatures or increase of temperature in the range of 2–4 °C is able to affect booting and flowering of rice and therefore affect rice yield. In addition to high temperatures, humidity also plays a role in spikelet sterility. However, there is significant difference in the tolerance of cultivars to high temperatures. For a variety to be temperature tolerant it would need to flower in cooler temperatures, have a higher viable pollen load, possess larger anthers and basal dehiscence. In addition to the structural modifications, these varieties should also have supply of protective proteins and enzymes that will protect rice against extreme temperature.

Exogenous application of SA has been implicated in the adaptive measures that help mitigate yield reduction in rice [68,69]. SA has also been shown to increase tolerance to heat in plants. Studies have been conducted to observe and assess heat tolerance in rice at various stages of development and the effect of SA in improving thermo-tolerance. As a consequence, it has been shown that heat shock affects seedling growth, biochemical activities, and mineral content. Further treatment with SA increased fresh and dry weight biomass in both resistant and susceptible rice lines, hence showing an overall increase in organic and inorganic solutes in rice plants [70]. Besides, when rice seedlings were treated with SA, there was no significant difference observed in pollen viability and seedling rate. When heat stress was introduced, however, SA reduced the levels of ROS in the anthers and thereby prevented programmed celled death of the tapetum. Tapetum associated genes, *EAT1* (Eternal Tapetum 1), *MIL2* (Microsporeless 2), and *DTM1* (Defective Tapetum and Meiocytese 1) showed elevated expression when treated with SA under heat stress. The pollen viability of rice with SA treatment was restored under heat stress. In addition, there was a sharp increase in H_2_O_2,_ which is important in mediating SA elicited prevention of pollen abortion in heat stress [71]. Rice seed germination is affected by temperature changes and SA concentration, where this exerts an effect on germination rate, biomass, root/shoot, and vigor index of root and shoot. Higher levels of SA are required when there is a large temperature drop i.e., from 30 to 15 °C. Pouramir et al. [72] showed that when rice seeds were imbibed in SA at 0, 20, 50, and 100 mg L^−1^ for 24 h and exposed to normal and chilling temperatures, root, shoot and emergence percentage were elevated in treated seeds. As in heat condition, chilling treatment also showed elevation of antioxidant enzymes in primed seeds [72].

High temperatures during flowering stage induce spikelet fertility in rice. If high temperatures were observed on flowering day during anthesis time, this would be most detrimental to spikelet fertility. However high temperatures post anthesis had little influence on spikelet fertility. In most cases spikelet fertility is caused by decreased viability of pollen grains resulting in drop in pollen grains [73]. In a study by Mohammed and Tarpley [74] the effect of SA on rice spikelet in panicles was determined at night temperatures around 27–32 °C. When treated with SA, the antioxidant activity within the spikelet was increased and this prevented membrane damage and protected the spikelet from undergoing any yield loss. Zhang et al. [75] reported that SA alleviated damage caused by heat stress on spikelet. Rice plants subjected to SA at 40 °C resulted in higher grain yield, spikelet number per panicle and setting rate. This resulted in higher soluble sugar content in plants, increased levels of proline, phytohormones and antioxidants. The compounds were higher in the spikelet with SA treatments compared to control and non-stressed conditions

Low temperatures can cause chilling injury that can affect so many processes within the plant including photo-inhibition. However, there has been no direct correlation between SA treatment and chilling tolerance. In fact, other than a slight endogenous increase in SA acid levels, rice plants did not respond positively to the treatment; exogenous SA seemed to result in reduction of chilling tolerance in rice [76]. While studies have shown that low temperature increases SA/oHCA levels in all plants including cereals, it would seem that SA was not an efficient secondary signal in eliciting a defense response to chilling in rice. Polyamines (PA) such as Putrescine (Put), Spermidine (Spd) and Spermine (Spm) have been shown to fluctuate in response to stress. Under cold stress PUT showed a slight increase in genotypes tested [77]. As there was no clear significant increase in SA in rice through stress, it may be concluded that PA and SA levels are regulated independently in rice and no clear positive implications can be drawn between SA and cold stress.

### 2.4. SA’s Effect on Metal Toxicity

Due to the use of chemical fertilizers, and pesticides, metal toxicity has become commonplace in fields and plantations. While there are several metals that can be observed in the soil as a consequence of chemical poisoning of soil, two metals have been extensively studied which are cadmium and arsenate. It has been reported that metals such as cadmium and arsenate can cause an increase in GPX activity while reducing GST activity indicating that there was no detoxification of lipid peroxidation in exposed rice (*Oryza sativa* L. cv. BRRI dhan54) plants [78].

In a study conducted in rice, the application of SA reversed the Cd induced effect. There are several hypothetical explanations on SA’s influence on Cd based on studies in maize. They are that (i) SA prevents cellular and membrane level damage caused by Cd [79], (ii) SA alleviates oxidative damage, and (iii) SA provides protection on membrane stability via lipid accumulation. Cd’s effect is mostly seen in the roots, as this is the first organ that is exposed to heavy metals. Heavy metal accumulation starts here and then it is translocated to the shoot. SA reduces the accumulation in organs and adds the benefit of better growth and photosynthesis. This postulates the possibility that the SA treatment is likely to reduce toxicity and manage stress through the activation of the antioxidant systems and lipid metabolism [80].

SA has been predicted to control Cd translocation to rice grains. Experiments show that SA reduced Cd transport from the stem to the leaf, and from the leaf to the panicles and grains, during the flowering stage of rice. This results in lower Cd accumulation in rice grains. Although some studies claim that SA has no effect on Cd accumulation in roots, stems, or nodes, with the exception of Cd levels in leaves at flowering, others claim that SA plays a role in Cd translocation from the root to the shoot system [81,82,83]. Contrary to this, SA was reported to have caused Cd accumulation in roots while inhibiting the translocation to the shoot in rice [82]. The differences observed on different plants is understandable as this may be attributed to the endogenous SA levels, but observations that vary within the same species may more likely be due to dose effect and method of SA application.

When SA and nitric oxide (NO) were used, there may be a cooperative defense activated in rice against Cd accumulation and stress. Taken together, this result implies that SA and NO may affect GPX-GST equilibrium and by so doing adjust tolerance and detoxification to excessive Cd [84]. SA and NaSA were also able to defend against Cd toxicity. NaSA and SA were able to afford different levels of protection against rice. This is probably due to their different effects on the activation of the antioxidant systems. It has been reported that the SA is involved in the regulation of Cd to leaves while NaSA increases phenolic compound (PC) levels in the roots. While there is no clear indication as to how these regulate Cd toxicity, it is postulated that the variation in their ionic strength (one acid, one salt) facilitates different reaction in plants [85].

Arsenate toxicity has also been studied in plants, where stressed plants showed high antioxidant enzymes such as CAT, SOD and ascorbate peroxidase (APX) levels believed needed to deal with the hydrogen peroxide content in cell. Furthermore, SA inhibits CAT and APX activity in rice [86]. There are contrasting views on how SA inhibits CAT where either SA chelation of Fe and/or through a peroxidation reaction that inhibits CAT [86]. Various isoforms of peroxidases have been reported in rice and have the ability to metabolize H_2_O_2_. Guo et al. [50] reported that arsenate enhanced GPX activity in a dose dependent manner in rice. There are also contradictory findings with regard to the effect of SA treatment to the levels of endogenous SA. Rice has high levels of endogenous SA and therefore does not show a marked increase in endogenous SA levels.

To shed some light on the mechanism of inhibition imposed by SA on metal transport within rice, studies were conducted on transporter genes (*OsLCT1* and *OsLCD*) that have been implicated in Cd transport via phloem [87]. The expression profiles of these transporter genes led to the postulation of three possible mechanisms by which toxic metal accumulation can be reduced in planta via SA. They are (i) metal accumulation post SA could lead to excess metal being sequestered in root vacuoles and less to shoots, (ii) SA assisted in the sequestering of toxic metals to the leaves and into leaf vacuoles and, finally, (iii) SA resulted in the chelation of toxic metal and lowered overall levels of metals in all rice tissues [88].

### 2.5. SA’s Effect on Nutrient Deficiency

Unfortunately, while there are reports on the effect of SA on plant systems, specific research on the effect of SA on nutrient deficiency in rice has not been undertaken nor reported widely. One common source of nutrient deficiency in rice is of nitrogen deficiency. Using SA as a source to alleviate the stress brought about by N deficient has been studied in various crops. While the role played by SA in stress tolerance in plants is not clear, it would appear that SA achieves alleviation by controlling physiological and biochemical processes in plants [89]. Deus et al. [90], in the experimentation to study the effect of SA alone or in combination with Si on alleviating nutrient stress in rice, reported on how these compounds affected net CO_2_ assimilation rate, carbon content, lignin, transpiration, stoichiometric ratio, and grain yield. According to their observations, SA did not help rice with nitrogen deficiency. In N-deficient conditions, Si alone was able to increase rice yield.

**Figure 1:**
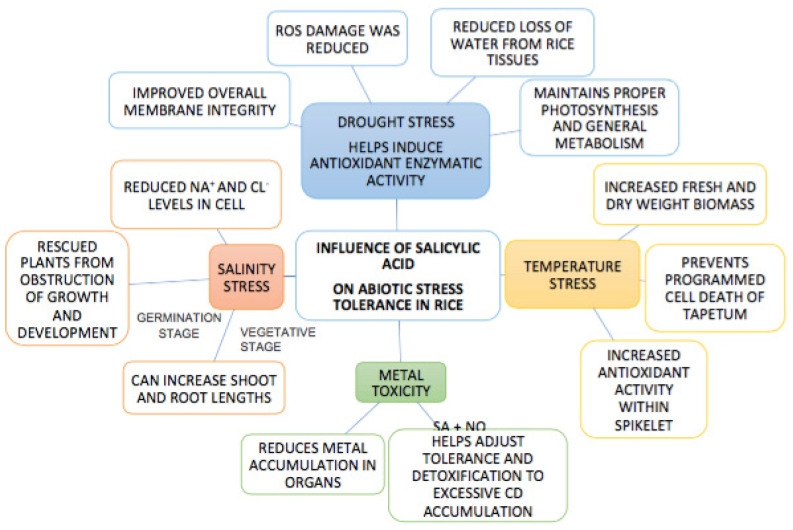
Schematic representation of the influences exerted by salicylic acid on rice when subjected to different abiotic stresses.

It is believed that the exogenous application of SA is able to reduce stress effect on plants. However, the effect of SA is greatly dependent on plant species, method of application, dose and environment. Therefore any application of SA below the threshold may not elicit any noticeable response in plants while amounts exceeding this threshold may have negative effects on the plant [91]. It is possible that the concentration used in the Deus et al. [90] study was insufficient to induce a response in rice. Further, SA’s influence is also controlled by species, phenotype, application method and the stress. Therefore, for any conclusions to be made on the SA–nutrient interaction, the dose, method of application, species, developmental stage and the level of stress has to be varied and monitored [90]. Figure 1 provides a diagrammatic representation as to how SA influences abiotic stress management in rice.

## 3. Mechanisms Regulating SA-Induced Stress-Tolerance

The effect that SA has on rice under environmental stresses is achieved through the interaction of SA with various components and mechanisms within the cell. In the following sections, these interactions are addressed.

### 3.1. SA Interacts with Osmolytes

Drought stress has its effect on plant osmolytes. SA application on drought stressed plants improved osmolyte regulation in planta. Studies have shown that there is an association between plant water relation and the buildup of solute such as proline within the plants. The presence of these solutes in rice helps maintain a low water potential that recruits compatible metabolites involved in osmoregulation and therefore ensures that there is sufficient water in the plant regardless of water shortage in the environment. Osmoregulation allows for absorption of more water from the surrounding to protect against water shortage within the plant [92].

In order to maintain osmotic turgor and to overcome abiotic stresses, plants accumulate osmolytes like proline, soluble sugars, glycine betaine, trehalose and others [93]. When the internal levels of proline, total soluble sugars (TSS), and betaine in plants are elevated, it enhanced stress tolerance [94,95]. An increase in TSS and proline levels was reported in Saryu-52 rice cultivar that was treated with biotic stresses and phytohormone treatments. From this study almost similar levels of proline were observed in the rice cultivar when treated with drought, salt, abscisic acid (ABA), SA, jasmonic acid (JA), and ethephon. This study also showed that phytohormones like ABA, SA, JA, and ethylene (ET) collectively had a role in moderating abiotic stress responses in various plants including rice [96]. Other phytohormones, however, may have a role to play in maintaining balance in growth and development (gibberellins, brassinosteroids, auxin, cytokinins, etc.) [97].

### 3.2. SA Facilitates Mineral Acquisition

Minerals are basic nutrient requirements of plants for their growth, development and survival. When mineral levels in plants are affected, growth and development is impaired and this results in alleviation of abiotic stress [16]. In abiotic stress, SA modulates nutrient acquisition to assist with growth and development [98]. In addition to the acquisition of mineral nutrients, SA plays a role in ensuring membrane integrity and regulation of trans-membrane flow [99]. Sharma et al. [100] had reported that SA played a crucial role in the uptake of elements such as Ca, Cu, Fe, Mn, P, and Zn. In case of heavy metal ions such a Pb, Co, Ni and Ar, SA mediated minimization of oxidative stress and protected the plant from damage to the photosynthetic organelle and the membrane [86]. SA-mediated stress management was observed in the presence of N, P, K, and Ca where supplementation of 0.5 mM SA improved growth, yield, gas exchange and salinity tolerance. This indicates the involvement of SA in moderating salinity tolerance in plants [44].

Nutrient uptake under individual and combined exposure to N, P, or K deprivation could be correlated to oxidative damage evident in rice seedlings. It was observed that the higher the level of ROS the higher the level of MDA content to cause oxidative damage. Similar results were also observed in Arabidopsis and wheat [101,102]. Further analyses were conducted to determine if seed priming did invoke an antioxidant defense mechanism with elevated stress enzymes. From here it was observed that with SA priming, higher SOD, CAT, POD, GR, and GSH activities were observed when nutrient was deprived. The higher content of antioxidants in primed rice seedlings is linked to lower levels of ROS in rice leaves. Previous studies on other cropping systems also showed that when seeds were primed, they increased antioxidant activities and therefore reduced ROS levels under drought stress [6].

### 3.3. SA Modulates ROS-Signaling and Antioxidant Activity

Abiotic and biotic stresses have both been reported to result in elevation of ROS. With environmental stresses, H_2_O_2_ begins to accumulate within the cell. These radicals have the ability to negatively affect the lipids, proteins and nucleic acids. Impacting these core components of the cell results in damage or impairment of normal cellular and physiological function [103]. The accumulation of ROS has been linked with damage to cellular and nuclear components resulting in electrolyte leakage, as a consequence of stress damage that can lead to deterioration. The remediation of ROS in plants is through scavenging via specific enzymes or molecules [104].

Treatment with SA results in the activation of scavenging enzymes like SOD and peroxidases. In certain plant systems CAT is induced. In some studies reduction in CAT activity was reported while others observed elevated levels of CAT [105,106]. For instance, CAT levels were inhibited with SA binding to CAT. This was not seen in rice. In tobacco the CAT readings may be from CATb activity, which is induced while CATa is inhibited [107].

The activation of the antioxidant system in rice post SA application was able to rescue the integrity of cell membrane and enable rice to retain its tissue water status, photosynthesis and physiological functions [108]. The NahG rice mutants, which are deficient in SA, produce elevated levels of ROS and low levels of antioxidants. The endogenous level of SA is also a contributing factor where the unusually high internal levels of SA seen in rice have been reported as a component responsible for modulating redox balance in rice and to protect it against oxidative damage. However SA was not able to successfully function as an effective secondary signal and activate the defense response in rice necessary to induce resistance [10].

In rice and also other cereals like maize, SA itself acts as an antioxidant that directly scavenges radicals and chelators where Fe-salicylate binds to SOD and facilitates the dismutation of superoxides [10]. Further, other than SA, related compounds like oHCA are able to induce abiotic stresses. This compound is also able to quench molecular oxygen in the cell. When drought tolerance was investigated in rice, GR enzyme activity was not affected though the resistant cultivars showed higher levels in leaves. The GR level is correlated to oHCA levels which is shown to function in a similar way to SA. oHCA was able to induce antioxidants in rice. Therefore not only SA, but SA like molecules may have a function in the plant acclimation mechanisms [55,56].

### 3.4. SA Influences Secondary Metabolites

All plants produce secondary metabolites that include substances including alkaloids, allinin, cyanogenic glucosides, defensins, glucosinolates, GSH, phenolics, non-protein amino acids, phytoalexins, terpenes, and thionins [44]. These metabolites have been rendered a role in biotic and abiotic resistance responses in plants [10]. Plants’ biotic and abiotic resistance responses have been linked to these metabolites [10]. SA has been linked to the development of secondary metabolites in various plant systems [109].

In looking into the metabolic regulation of secondary metabolites in rice over the past decades, we were able to determine that while some are regulated as in other plant species, some have a unique metabolic pathways in rice (i.e., diterpenoid phytoalexins) [110]. While a large number of secondary metabolites have been identified, their physiological functions have not been elucidated. The generation of null mutant and overexpression transgenic lines have been used to help with the identification of these secondary metabolite functions in rice [111]. However, we must acknowledge that rice secondary metabolite biosynthetic control is extremely complex. Therefore, the regulation of secondary metabolites requires a repertoire of regulators out of which SA is just one of the triggers. Further, considering that the endogenous levels of SA in rice (*Oryza sativa* L. cv. Nipponbare) are high and very rarely show increase in response to treatment, it is hypothesized that SA may not play that dominant a role in the regulation compared to JA [112].

In rice leaves and suspension-cultured cells, natural and synthetic CKs induced the synthesis of diterpenoid phytoalexins. In rice leaves, however, CK treatment inhibited the development of JA-inducible sakuranetin. Exogenous root applications of SA, on the other hand, facilitated the accumulation of oryzalexins and momilactone A in the leaves. A synergistic crosstalk of CK and SA signaling was also discovered, showing that combining 0.1 mM CKs with benzothiadiazole (BTH), a plant activator that enhances the SA signaling pathway, increased momilactone and phytocassane biosynthetic genes by several fold [113]

### 3.5. SA and Hormones

Via cross-talk with other phytohormones, SA has been shown to play an important role in the control of stress and nonstress conditions [69]. SA’s interaction with auxin, cytokinin, gibberellin, abscisic acid, ethylene, nitrous oxide and brassinosteroids has been studied in various plant systems in stress and nonstressed systems [114,115]. SA’s interactions with other phytohormones may be synergistic or antagonistic. For instance, it would appear that SA worked antagonistically with auxin to moderate stress response [116]. Meanwhile SA has been reported to trigger ABA accumulation under salinity stress and this initiates an osmotic adaptation response in the plant that protects the photosynthetic machinery among others. Together with SA, ABA also regulates cold response where in *Z. mays*, ABA improved endogenous SA and oHCA leading to speculation that there may be cross or shared regulation of SA related stress responses with ABA [44]. Further, ethylene has been shown to be actively involved in environmental stresses where it controls the effects of stress through oxidative stress management [93] in an antagonistic manner. However, according to Ghanta et al. [117] there are also examples where the interaction between ethylene and SA are synergistic in environmental stresses. In addition to the above, NO has been reported to work as a secondary messenger with SA in regulating stress in response to environmental stresses [118]. While working downstream from SA, NO moderates abiotic stress via control over oxidative stress [12]. In various studies, SA, JA and various other phytohormones have been reported and their roles in biotic and abiotic stresses elucidated.

**Figure 2:**
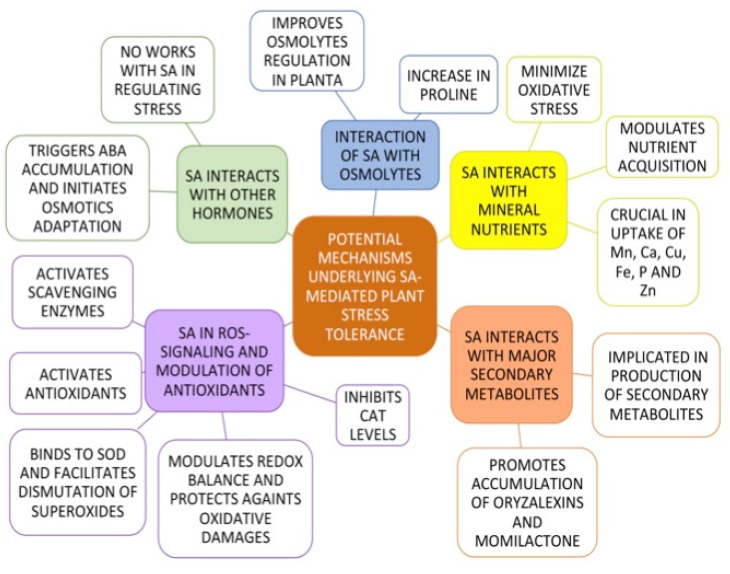
A schematic representation of the mechanisms utilized by SA in regulating stresses in rice.

When we start elucidating the SA pathway in rice, there are some master regulators that may be shared with other plants species. One such regulator is the NPR1. NPR1 is known to be translocated from the cytosol into the nucleus to interact with transcriptional factors that will activate defense related genes. NPR1 and its paralogs, NPR3 and NPR4 serve together with SA receptor proteins to activate plant responses to stress [119,120]. In Arabidopsis almost all BTH responsive genes are controlled by NPR1. However, in rice (*Oryza sativa* L. cv. Nipponbare) another regulator, OsWRKY45, regulates a large number of these genes. The involvement of OsWRKY45 in rice SA signaling has not been clearly understood [121]. However, Ueno et al. [122] indicated that OsWRKY45 may be activated by a SA dependent phosphorylation through a MAPK controlled pathway. Most of the studies thus far have implicated that both OsNPR1 and OsWRKY45 regulate the SA pathway in rice. Nakayama et al. [123] in their microarray experiment showed that there were BTH responsive genes that were up and downregulated in rice. The downregulated genes controlled by BTH is regulated by OsNPR1, which relocates energy and resources by downregulation of processes such as photosynthesis and other protein processes. However, OsWRKY45, which controls well-known genes and regulators including OsWRKY62, OsNAC4, and OsHSF1, controlled the upregulated BTH sensitive genes [123,124]. These findings suggest that OsNPR1 and OsWRKY45 play complementary roles in the rice SA pathway in response to environmental stresses. Figure 2 provides a diagrammatic representation on the mechanisms utilized by SA in regulating stresses in rice.

## 4. Conclusions and Future Prospects

Significant progress has been made in the discovery and characterization of a number of SA signaling components involved in a variety of cellular processes over the last two decades. Recent SA-centered discoveries have significantly increased the capabilities of this molecule beyond its previously identified functions in local and systemic defenses, development, stress management, cellular repair, growth, senescence and programmed cell death. In the recent years SA research has entered a new phase where the intricacies of the SA-mediated signaling processes in growth, development and stress management has been elucidated under various environmental conditions given to the drastic changes in environment observed with climate change. While much may have been done in plant species to understand SA involvement in biochemical and physiological processes, little remains known on rice. Some areas that require further focus and research are (1) to completely elucidate SA biosynthesis in rice and to address the redundancy in the pathways involved. In addition, the expression levels and nature of the cross-talk between the different components of these two pathways must be further dissected; (2) to obtain a better understanding as to the mechanism of SA perception and interaction with other hormone networks and to identify signal(s) that may regulate various biological and physiological processes including stress management in rice; (3) to further dissect the influence that SA has on processes in rice and to determine clearly the mechanism of interaction; (4) finally, additional genetic studies pertaining to SA sensitivity could unravel novel molecules that would increase our understanding of the SA pathway in rice and the reason behind the higher levels of SA in rice. Further research on the multifaceted functions of SA will greatly advance our nuanced understanding of the molecular underpinnings of rice abiotic stress management. More importantly this information will provide us with a better understanding of rice defense mechanism and to utilize the information in resistance breeding and exogenous induction of defenses in rice modern agriculture.

## Data Availability

Not applicable.
